# Zn(II)-Chlorido Complexes of Phytohormone Kinetin and Its Derivatives Modulate Expression of Inflammatory Mediators in THP-1 Cells

**DOI:** 10.1371/journal.pone.0065214

**Published:** 2013-06-03

**Authors:** Jan Hošek, Radka Novotná, Petr Babula, Ján Vančo, Zdeněk Trávníček

**Affiliations:** 1 Department of Inorganic Chemistry, Regional Centre of Advanced Technologies and Materials, Faculty of Science, Palacký University, Olomouc, Czech Republic; 2 Department of Natural Drugs, Faculty of Pharmacy, University of Veterinary and Pharmaceutical Sciences Brno, Brno, Czech Republic; University of Strathclyde, United Kingdom

## Abstract

Kinetin (N6-furfuryladenine) belongs to a group of plant growth hormones involved in cell division, differentiation and other physiological processes. One of the possible ways to obtain biologically active compounds is to complex biologically relevant natural compounds to suitable metal atoms. In this work, two structural groups of Zn(II) complexes [Zn(L^n^)_2_Cl_2_]·Solv (**1**–**5**) and [Zn(HL^n^)Cl_3_]·*x*L^n^ (**6**–**7**); n = 1–5, Solv = CH_3_OH for **1** and 2H_2_O for **2**; *x* = 1 for **6** and 2 for **7**; involving a phytohormone kinetin and its derivatives (L^n^) were evaluated for their ability to modulate secretion of tumour necrosis factor (TNF)-α, interleukin (IL)-1β and matrix metalloproteinase (MMP)-2 in a lipopolysaccharide (LPS)-activated macrophage-like THP-1 cell model. The penetration of the complexes to cells was also detected. The mechanism of interactions of the zinc(II) complexes with a fluorescent sensor *N*-(6-methoxy-8-quinolyl)-*p*-toluene sulphonamide (TSQ) and sulfur-containing biomolecules (l-cysteine and reduced glutathione) was studied by electrospray-ionization mass spectrometry and flow-injection analysis with fluorescence detection. The present study showed that the tested complexes exhibited a low cytotoxic effect on the THP-1 cell line (IC_50_>40 µM), apart from complex **4**, with an IC_50_ = 10.9±1.1 µM. Regarding the inflammation-related processes, the Zn(II) complexes significantly decreased IL-1β production by a factor of 1.47–2.22 compared with the control (DMSO), but did not affect TNF-α and MMP-2 secretions. However, application of the Zn(II) complexes noticeably changed the pro-MMP-2/MMP-2 ratio towards a higher amount of maturated MMP-2, when they induced a 4-times higher production of maturated MMP-2 in comparison with the vehicle-treated cells under LPS stimulation. These results indicated that the complexes are able to modulate an inflammatory response by influencing secretion and activity of several inflammation-related cytokines and enzymes.

## Introduction

Cytokinins form a group of plant hormones, which take part in regulation of all stages of plant growth represented by different cellular processes, including cell division, control of leaf senescence, control of nutrient allocation, root nodule development, stem cell maintenance, and regulation of auxin action [1]. Kinetin (N6-furfuryladenine) is classified among naturally occurring cytokinins [2] and since its isolation and identification, kinetin has been widely used in various aspects of plant research, including applications in biotechnology and cell biology mainly due to its stimulation on plant growth, retardation of leaf senescence and modulation of response of plants to various environmental stresses. In addition to the regulatory functions in plants, kinetin has also showed strong anti-ageing activity on different animal targets, such as fruit flies [3], or human dermal cells [4], [5]. These positive effects led to the commercial application of kinetin in rejuvenating medicinal cosmetics [6], [7].

In the last few years, it is also possible to observe significant accrual of studies, focused on investigation of different effects of phytohormones (such as zeatin riboside, kinetin, or abscisic acid) and their derivatives on human cell division and dominantly the ability of these natural compounds to intervene into the proliferation of different human cancer cell lines [8]–[10]. The substantial antiproliferative effects of some cytokinins were established and the studies performed so far indicate, that this direction in research could lead to identification of potent bioactive compounds with relevant anti-cancer activity.

In both above mentioned applications (anti-tumour and anti-aging) of cytokinins, including kinetin and its derivatives, the immunomodulating ability and the influence on inflammatory processes play very important roles [11]–[13]. To the best of our knowledge, there are only three publications evaluating the inflammatory-modulating effects of kinetin to date. Two of these records describe the anti-inflammatory effect of kinetin and other phytohormones (e.g. *trans*-zeatin and N6-benzyladenine) applied in 1% (w/v) topical preparations for the treatment of mild to moderate cases of inflammatory rosacea [14] or other inflammatory skin conditions that manifest themselves as skin lesions [15]. The third record [16] provides data from an *in vivo* study on rats exposed to the plant growth regulators indolacetic acid and kinetin delivered in drinking water. In this study, the activities of antioxidant and immune marker enzymes were studied. In contrast to the studies on topically applied kinetin, this study provides evidence that several antioxidant enzymes were inhibited by the kinetin exposure leading to the presumption that this condition could facilitate the process of inflammation.

One of the possible ways to obtain biologically active compounds with unique pharmacological properties is complexing of biologically relevant natural compounds, very often of plant origin, to suitable metal atoms. This approach can lead to substances which can exert a different mode of interaction with the organism in connection with the possible synergistic effect of the metal ion and organic molecule, as we demonstrated in the case of anti-inflammatory effects of gold(I) complexes with derivatives of cytokinin N6-benzylaminopurine [17]. The recent results concerning a zinc(II) complex involving curcumin can also be named as a successful fulfillment of such a concept as the compound demonstrated a better antiphlogistic effect than curcumin alone [18].

Zinc is classified among “elements essential for higher animals” [19]. Due to key roles of zinc in many fundamental biochemical processes, abnormal zinc homeostasis is related to varied health problems including growth retardation, neuronal dysfunctions and cancer [20]. Zinc deficiency is involved in higher susceptibility to infection and increases the pro-inflammatory status [21]–[22]. Several articles show that, depending on the experimental conditions and biological target system, zinc could act either as a pro-inflammatory factor due to the activation of the transcription factor NF-κB [23]–[25], or more frequently as an anti-inflammatory factor via different biochemical pathways, such as (i) the mutual inhibition of the oxidative stress and pro-oxidative enzymes (e.g. NADPH oxidase), (ii) the induction of anti-oxidative defence systems (e.g. increasing production of metallothioneins, superoxide dismutase), and (iii) the inhibition of the NF-κB transcription factor (zinc causes zinc-finger protein up-regulation and the inhibition of the NF-κB activation through a TRAF pathway), resulting in the reduction of inflammatory cytokines and adhesion molecules [26]–[28]. Several zinc(II) complexes were also previously tested on different inflammatory models and showed significant diminution of induced inflammation [29]–[31].

On the basis of the documented biological activities of cytokinins and zinc immune modulating activity, we decided to test previously prepared and described Zn(II) complexes involving kinetin and its derivatives [32], [33] for their anti-inflammatory activity on an *in vitro* cell model. To the best of our knowledge, the ability of kinetin or its derivatives to modulate inflammatory signal pathways has not been studied yet and thus this study represents a completely novel approach with unique results.

We focused on the production of typical pro-inflammatory cytokines such as tumour necrosis factor (TNF)-α and interleukin (IL)-1β and inflammatory-related matrix metalloproteinase (MMP)-2 in this study. The ability of these compounds to penetrate cells was also studied as well as the mechanism of interactions with a fluorescence probe and sulfur-containing molecules.

## Materials and Methods

**Figure 1:**
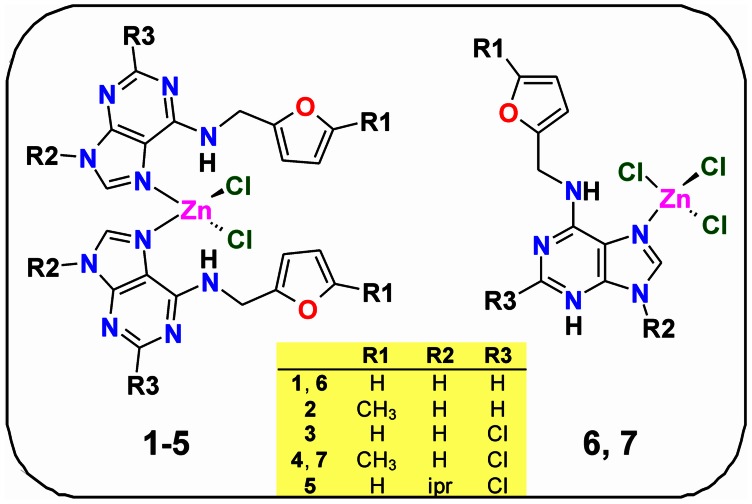
Schematic representations of complexes 1–7.

All the chemicals and solvents were purchased from commercial sources and were used as received. The syntheses and characterizations of the Zn(II) complexes were reported previously [32], [33]; the complexes [Zn(L^1^)_2_Cl_2_]·CH_3_OH (**1**), [Zn(L^2^)_2_Cl_2_]·2H_2_O (**2**), [Zn(L^3^)_2_Cl_2_] (**3**), [Zn(L^4^)_2_Cl_2_] (**4**), [Zn(L^5^)_2_Cl_2_] (**5**), [Zn(HL^1^)Cl_3_]·L^1^ (**6**), and [Zn(HL^4^)Cl_3_]·2L^4^ (**7**) involve kinetin (L^1^) and its derivatives, N6-(5-methylfurfuryl)adenine (L^2^), 2-chloro-N6-furfuryladenine (L^3^), 2-chloro-N6-(5-methylfurfuryl)adenine (L^4^) and 2-chloro-N6-furfuryl-9-isopropyladenine (L^5^) as N-donor ligands (Figure 1).

### Monocyte Cultivation and Cytotoxicity Determination

For the cytotoxicity measurements, we used the human monocytic leukemia cell line THP-1 (ECACC, UK). The cells were cultivated at 37°C in RPMI 1640 medium supplemented with 2 mM of l-glutamine (Lonza, Belgium), 10% (v/v) FBS (Sigma-Aldrich, Germany), 100 U/mL of penicillin and 100 µg/mL of streptomycin (Lonza, Belgium) in a humidified atmosphere containing 5% CO_2_. Stabilized cells (3^rd^–15^th^ passage) were split into 96-well microtitre plates to a concentration of 500 000 cells/mL.

The measurements were taken 24 h after the treatments with 6.25, 12.5, 25, 50 or 100 μM of the tested compounds dissolved in dimethyl sulfoxide (DMSO) [the final DMSO concentration was 0.1% (v/v)]. Viability was measured by the WST-1 test (Roche, Germany) according to the manufacturer’s manual. The amount of created formazan (correlating to the number of metabolically active cells in the culture) was calculated as a percentage of control cells (treated only with DMSO) and was set as 100%. The cytotoxic IC_50_ concentrations of the compounds were calculated by the GraphPad Prism 5.02 (GraphPad Software Inc., San Diego, CA).

### Differentiation to Macrophages

To determine the influence of the tested complexes on the TNF-α and IL-1β secretions and MMPs activity, macrophage-like cells derived from the THP-1 cell line were used. The cells were cultivated as above, but were split into 24-well microtitre plates to get a concentration of 100 000 cells/mL (1 mL/well) and the differentiation to macrophages was induced by phorbol myristate acetate (PMA) as described previously [34].

### Treatment with Complexes and Induction of Inflammatory Response

Differentiated macrophages were pre-treated for 1 h with 5 µM solutions of the tested complexes or 1 µM prednisone dissolved in DMSO [the final DMSO concentration was 0.1% (v/v)] and with the 0.1% (v/v) DMSO solution itself (the experimental group called *vehicle*); the given concentrations of the compounds lack the cytotoxic effect. The inflammatory response was triggered by adding 1 µg/mL *Escherichia coli* 0111:B4 lipopolysaccharide (LPS) (Sigma-Aldrich, Germany) dissolved in water to pre-treated macrophages, *control* cells were without the LPS treatment. Each experiment was performed in triplicate.

### Evaluation of Cytokine Secretion by ELISA

Macrophages, which were pre-treated with the tested compounds for 1 h, were incubated with LPS for the next 24 h. Then, the medium was collected and the concentrations of TNF-α and IL-1β were measured by an Instant ELISA kit (eBioscience, Austria) according to the manufacturer’s manual.

### Zymography

The conditioned media obtained in the same way as for cytokine evaluation were used for the measurement of MMP-2 and MMP-9 activity by zymography. 30 μL of the collected medium stained with a loading dye [250 mM Tris-HCl pH 6.8, 10% (w/v) SDS, 30% (v/v) glycerol, 0.04% (w/v) bromphenol blue] in a ratio of 4∶1 was loaded into a 10% (w/v) polyacrylamide gel impregnated with 0.1% (w/v) gelatin. After electrophoresis, SDS from the gels was washed out with 2.5% (v/v) Triton X100 and the gels were incubated for 30 minutes at room temperature (∼ 23°C) and subsequently overnight (16–20 hours) at 37°C in a developing buffer [50 mM Tris-HCl pH 8.8, 5 mM CaCl_2_, 3 mM NaN_3_, 0.5% (v/v) Triton X100]. The gels were stained with Coomassie brilliant blue R-250 (Amresco, USA) [0.1% (w/v) Coomassie brilliant blue R-250 was dissolved in the mixture of 50% (v/v) methanol and 10% (v/v) acetic acid in water]. Subsequently, the gels were moderately destained with the mixture of 50% (v/v) methanol and 10% (v/v) acetic acid in water. The intensity of the digested regions was calculated by AlphaEasy FC 4.0.0 software (Alpha Innotech, USA) for densitometric analysis.

### Zinc Cellular Uptake

For fluorescence microscopy, the THP-1 macrophages were cultivated on LAB-TEK chamber slides (made from Permanox plastic). After 4 and 24 hours of incubation with the tested complexes and ZnCl_2_ (served as a control) at the final concentration of 5 µM, the microscope slides with a monolayer of the cells were rinsed with the cultivation medium without the zinc(II) complexes and PBS buffer and directly used for staining and fluorescence microscopy. For zinc(II) staining, a fluorescent probe *N*-(6-methoxy-8-quinolyl)-*p*-toluene sulphonamide (TSQ, Invitrogen, USA) was used. The working solution (10 mM phosphate buffer pH 7.6) was prepared by diluting the TSQ stock solution (10 mM, acetone). The cells were carefully rinsed with PBS buffer to remove all cultivation media containing the residues of the tested compounds, and subsequently stained with a working TSQ solution (30 min, 37°C, dark), washed with PBS buffer (pH 7.6) and observed under a fluorescence microscope (Axioskop 40, Carl Zeiss, Germany) equipped with FITC and DAPI filters (Carl Zeiss, Germany). Photographs were taken using a digital camera (Olympus Camedia 750, Olympus, Japan). NIS elements software (Nikon, Japan) was used for the evaluation of the relative intensity, which reflects the levels of zinc(II) species. All samples were evaluated in triplicates, 10 random fields were evaluated.

### Mass Spectrometry and Fluorescence Spectra Measurements

To describe more precisely the mechanism of interaction of zinc(II) complexes with TSQ (used in the cell-uptake studies) and sulfur-containing biomolecules (l-cysteine, reduced glutathione), the electrospray-ionization mass spectrometry (ESI-MS) and flow-injection analysis with fluorescence detection (FIA-FLD) were used. The conditions for the ESI-MS analysis were published recently [35]. The reactions of the representative zinc(II) complex **5** with TSQ were performed in methanol, while the solutions of l-cysteine and glutathione (at the final concentrations of 290 μM, and 6 μM [36], respectively) dissolved in 50 mM of ammonium acetate in water were studied in the mixture with complex **5** dissolved in methanol (1∶1, v/v). The FIA-FLD system was composed of the different parts of HP 1100 HPLC system (Agilent Technologies, Germany), namely the quaternary pump, degasser, autosampler, column thermostat, and fluorescence detector using the excitation wavelength of 365 nm and the emission spectra were collected in the range from 400 to 900 nm; flow rate of methanol was set to 0.1 mL/min, and all solutions were heated up to 37°C.

### Statistical Analysis

All experiments were performed in three independent experiments; the results are presented as mean values, with error bars representing the standard error (S.E.) of the average value. A one-way ANOVA test was used for the statistical analysis, followed by a Tukey’s *post-hoc* test for multiple comparisons. A value of *P<*0.05 was considered to be statistically significant. GraphPad Prism 5.02 (GraphPad Software Inc., San Diego, CA) was used to perform the analysis.

## Results and Discussion

Zinc represents an important microelement in living systems. On the basis of its natural antiphlogistic and antioxidant activities, seven Zn(II) complexes were tested *in vitro* for these effects.

First of all, the cytotoxic effect of the tested compounds was determined. Low toxicity was expected, because the tested complexes involve two (**1**–**5**) or one (**6**–**7**) coordinated N-donor kinetin moieties and its derivatives, which can positively influence cell metabolism and proliferation (see current review [2]), and a relatively non-toxic zinc(II) ion [20]. This expectation was fulfilled for all the complexes, except for **4**, which demonstrated an IC_50_>40 µM. Only **4** showed a slight toxic effect with an IC_50_ = 10.9±1.1 µM. We cannot explain this phenomenon yet, however, it is possible, that **4** acts as a cyclin-dependent kinase inhibitor, in a similar way as the structurally resembling Zn(II) complexes with the derivatives of N6-benzyladenine [37]. Nevertheless, the interaction between **4** and its target protein has to be very specific, because structurally very similar complexes **1**–**3** and **5** did not demonstrate this effect. On the basis of these results, the concentration of 5 µM was used for all the following experiments, because the solutions of the complexes of such concentrations lack any cytotoxic effect. The free ligands L^1^– L^5^ did not affect cell proliferation themselves at the used concentration of 5 µM.

**Figure 2:**
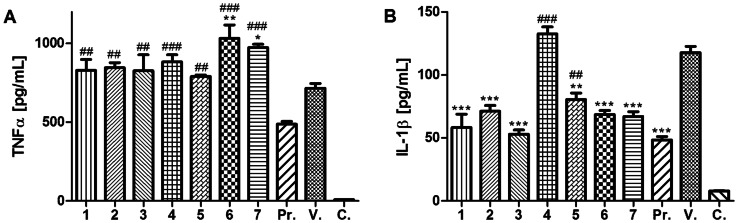
Effects of Zn(II) complexes and drug prednisone on LPS-induced TNF-α (A) and IL-1β (B) secretion. The cells were pre-treated with complexes **1**–**7** (5 µM), prednisone (**Pr.**, 1 µM) or the *vehicle* (**V.**, DMSO) only. After 1 h of incubation, the inflammatory response was induced by LPS [except for the *control* cells (**C.**)]. The secretion was measured 24 h after LPS addition. The results are expressed as means ± S.E. for three independent experiments. Significant difference in comparison to: * vehicle-treated cells (*P<*0.05), ** vehicle-treated cells (*P<*0.01), *** vehicle-treated cells (*P<*0.001), ## prednisone-treated cells (*P<*0.01), ### prednisone-treated cells (*P<*0.001).

To evaluate the antiphlogistic potential of the given zinc(II) complexes, the expression of pro-inflammatory cytokines TNF-α and IL-1β was measured in the LPS-stimulated macrophage-like cells THP-1. The previously reported results indicated the potential of zinc(II) ions to inhibit the expression of TNF-α, at least partly, due to the inhibition of the IKK (IκB kinase; important for NF-κB activation) activity [38] and/or activation of A20 (zinc-finger transactivating factor; inhibitor of NF-κB) [39]. However, as it is obvious from Figure 2A, the results of this study did not confirm the ability of the tested compounds to attenuate TNF-α production. Complexes **1**–**5** moderately non-significantly increase (1.10–1.23 times in comparison with the vehicle-treated cells) secretion of TNF-α, while compounds **6** and **7** even significantly augmented its expression by a factor of 1.44, and 1.36, respectively. However, the complexes alone (without LPS activation) were not able to induce TNF-α secretion. Our observation corresponds with the different structural arrangements of the compounds **6** and **7** with respect to **1**–**5**. Complexes **6** and **7** involve the ZnCl_3_ moiety whose charge is compensated by the coordination of one N3-protonated organic ligand HL^1^ or HL^4^ [32]–[33]. Additionally, the organic molecules out of the coordination sphere of the metal might have an influence on the results of this testing. Still, the effect on TNF-α levels was marginal, and the physiological relevance of the augmentation should be tested on *in vivo* models in detail.

Generally, the inability to attenuate the TNF-α secretion could be caused by the lower concentration used in this experiment (5 µM in this study, 15 µM Zn^2+^ in the study of Prasad *et al*. (2011) [39] and even 120 µM in the study of Jeon *et al*. (2000) [38]) and thus by insufficient inhibition of LPS stimulation. Additionally, a different cell line was investigated in our study - Jeon *et al*. (2000) [38] used the murine RAW264.7 cell line and Prasad *et al*. (2011) [39] human HL-60 and HUVEC cell lines. As described in the paper of von Bulow *et al*. (2005) [40], the concentration of zinc could dramatically influence TNF-α expression. Lower concentrations of this metal increased TNF-α production, while higher concentrations reduced it. Also another explanation for how TNF-α secretion remains unchanged could be due to, e.g. higher activity of TACE (tumour necrosis factor-α-converting enzyme), which converts membrane-bound TNF-α (mTNF-α) to its soluble form (sTNF- α). Zinc-dependent matrix metalloproteinase TACE is the only known enzyme, which converts mTNF-α to sTNF-α, thus its activity is rate-limiting for secretion of this protein. Recently, it has been described, that the THP-1 cell line, catalytically changes TACE activity after LPS activation [41]. It is possible that the tested zinc(II) complexes fixed TACE active conformation and thus support TNF-α shedding.

Unlike TNF-α, the expression of the second pro-inflammatory cytokine IL-1β was significantly attenuated by the tested complexes (Figure 2B). All the complexes, except for **4**, significantly decreased IL-1β secretion by a factor of 1.47–2.22 (in comparison with the vehicle-treated cells). These values were similar to prednisone, which attenuated the expression of this cytokine 2.42-times. This finding is in concordance with the previously reported experiments, where the reduction of IL-1β production was observed in the LPS-stimulated cells after the application of zinc [39], [40]. The only exception was represented by complex **4**, which slightly increased secretion of this cytokine by a factor of 1.13. This could be explained by its different toxicity pattern in comparison with the other compounds. Probably, when the subtoxic concentration of **4** is used, it acts in a rather pro-inflammatory than anti-inflammatory way.

Expression of TNF-α and IL-1β is under control of the transcription factor NF-κB, thus up to transcription, they have similar regulation, but in the state of mRNA, their fates diverge, i.e., while mTNF-α is cleaved by zinc-dependent metalloenzyme TACE [41], the pro-IL-1β is hydrolysed by caspase-1 [42]. The tested complexes **1**–**7** demonstrated a diametrically different effect on the production of these cytokines; whilst TNF-α slightly increased, IL-1β significantly decreased. On the basis of this observation, it is possible to propose that these zinc(II) complexes act down-stream of the transcription step during the expression of TNF-α and IL-1β.

The individual contribution of kinetin derivatives to modulation of cytokine secretion remains unclear. There are only two clinical trials involving the use of kinetin in topical preparations for the treatment of inflammatory related skin conditions, which reported positive effects of this phytohormone on the clinical manifestations of the disease [14]–[15]. On the other hand, Celik *et al.* (2006) [16] speculated in their work about negative effects of kinetin on cell-mediated immunity. This discrepancy could be caused by different experimental models (human vs. rat) and/or different application forms (topical/peroral). Our data contribute to this dispute and offer a new perspective in connection with the effect of the essential transition metal zinc. We showed that the zinc(II) complexes of kinetin had different effects on the secretion of pro-inflammatory cytokines. However, a detailed *in vivo* study would be necessary to arbitrate this discrepancy.

**Figure 3:**
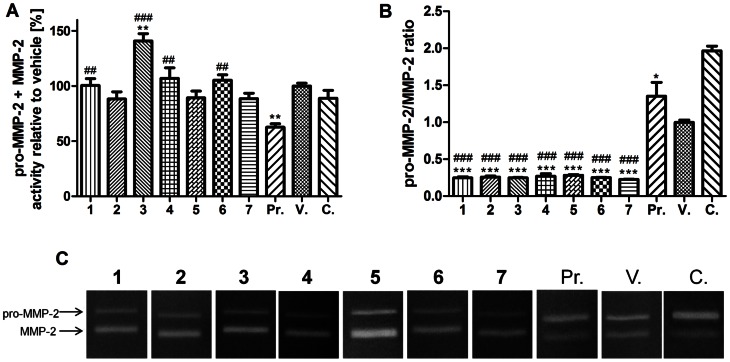
Effects of Zn(II) complexes and drug prednisone on LPS-induced matrix metalloproteinase 2 (MMP-2) activity (3A). The cells were pre-treated with complexes **1**–**7** (5 µM), prednisone (**Pr.**, 1 µM) or the *vehicle* (**V.**, DMSO) only. After 1 h of incubation, the inflammatory response was induced by LPS [except for the *control* cells (**C.**)]. Activity of MMP-2 was detected by zymography. The intensity of the digested bands was analysed by densitometric analysis. Fig. **3B** shows the pro-MMP-2/MMP-2 ratio. The results are expressed as means ± S.E. for three independent experiments. The gels show the representative results of three independent experiments (**3C**). Significant difference in comparison to: * vehicle-treated cells (*P<*0.05), ** vehicle-treated cells (*P<*0.01), *** vehicle-treated cells (*P<*0.001), ## prednisone-treated cells (*P<*0.01), ### prednisone-treated cells (*P<*0.001).

MMP-2 and MMP-9 were the last studied inflammatory-related proteins. They have a physiological function during tissue development and remodelling, but they also contribute to inflammation progression [43]. We found that the tested zinc(II) complexes did not affect the expression of MMP-9 (data not shown). Similar results were observed for MMP-2 (Figure 3A). All the complexes, except for **3**, showed only gentle influence on the MMP-2 amount (88–107% of vehicle-treated cells). Compound **3** significantly increased the secretion of this protein (141% of vehicle-treated cells). However, the ratio between physiologically inactive pro-MMP-2 and active MMP-2 (Figure 3B) is noteworthy. All the used complexes noticeably changed this ratio in the direction of a higher amount of MMP-2 and lower amount of pro-MMP-2. The complexes induced ∼ 4-times production of maturated MMP-2 in comparison with the vehicle-treated cells under LPS stimulation. A similar phenomenon was detected by de Souza *et al*. (2000) [44], where they tested direct inhibition of MMPs by ZnSO_4_. In this paper, a decrease of pro-MMP-2 activity at lower concentrations of zinc ions was followed by diminution of MMP-2 activity at higher concentrations of zinc ions, as is apparent from zymographs. We cannot explain the observed phenomenon yet. The maturation of pro-MMP-2 to MMP-2 is carried out predominantly by membrane type (MT)1-MMP. Also the presence of the right concentration of the tissue inhibitor of metalloproteinase (TIMP)-2 is necessary. Creating the TIMP-2/MT1-MMP/pro-MMP-2 complex is important for correct maturing of MMP-2. On the one hand, a low concentration of TIMP-2 is not sufficient for optimal pro-MMP-2 processing, on the other hand, a higher concentration of TIMP-2 inhibits MT1-MMP activity. Hence, the ratio among TIMP-2, MT1-MMP and pro-MMP-2 is crucial for MMP-2 activity [45]. However, pro-MMP-2 can also be activated in a TIMP-2-independent pathway by MT2-MMP [46] or by other mechanisms, e.g. by reactive oxygen species (ROS) [25]. It is possible, that the complexes **1**–**7** are involved in some of the above mentioned mechanisms of pro-MMP-2 activation.

**Figure 4:**
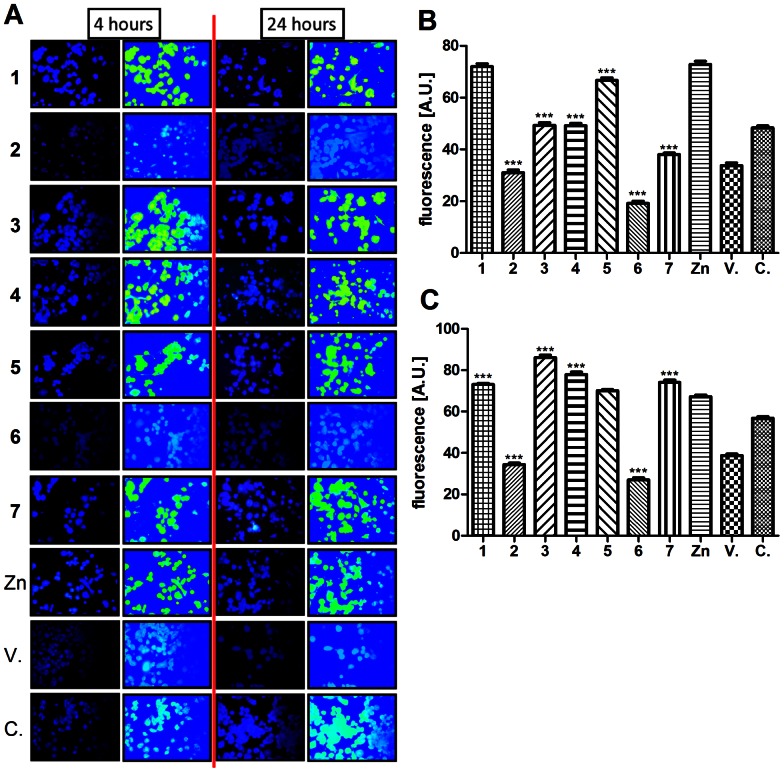
Zinc cellular uptake. THP-1 macrophages were incubated with tested complexes **1**–**7** (5 µM), ZnCl_2_ (5 µM), the *vehicle* (**V.**, DMSO) and compared with untreated cells [the *control* cells (**C.**)]. The Zn(II) cellular uptake was determined after 4 and 24 hours of incubation using *N*-(6-methoxy-8-quinolyl)-p-toluene sulphonamide. Obtained pictures (see first column for 4/24 hours, **A**) were subsequently processed, the concentration of zinc(II) ions was converted into a concentration colour scale. Relative intensity of emission was calculated for both treatments (see **4B** for 4 hour and **4C** for 24 hour incubation). Results are expressed as means ± S.E. for three independent experiments. *** significant difference in comparison to ZnCl_2_-treated cells (*P<*0.001).

To clarify whether the zinc(II) species from compounds **1**–**7** are able to penetrate cells, the cellular Zn(II) uptake was determined (Figure 4). DMSO (vehicle) slightly decreased the intracellular zinc contents, but the addition of Zn(II)-containing complexes dissolved in DMSO changed its amount in the cells. When the cells were exposed to the presence of **2** and **6**, the amount of intracellular zinc was even lower than after the DMSO treatment and this situation did not change after longer (24 hours) incubation (Figure 4C). Compounds **3**, **4** and **7** showed a lower cellular zinc uptake than ZnCl_2_ after 6 hours of the incubation, but they were comparable with ZnCl_2_ after 24 hours of incubation. Complexes **1** and **5** reached the maximal penetration after 6 hours and their amount did not change after 24 hours incubation. No correlation between the zinc complex cellular uptake and the production of the inflammatory-related cytokines and enzymes was observed. This is in agreement with the observation of Pavlica and Gebhardt (2010) [47], who found that the penetration of zinc(II) salts into cells did not correlate with their cytoprotective effects.

**Figure 5:**
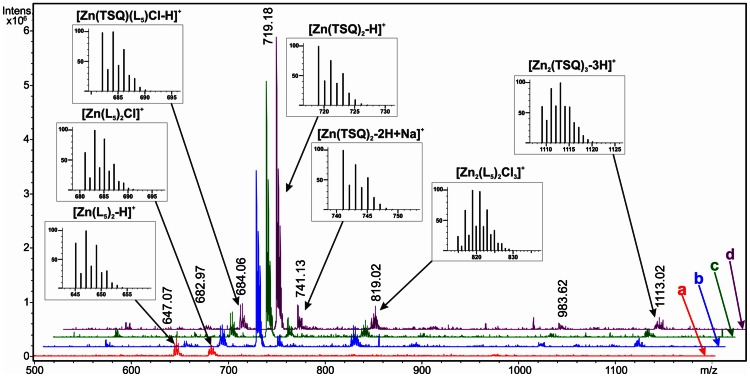
ESI-MS study of the mechanism of interaction of Zn(II) complexes with the used fluorescence probe. ESI-MS spectra of (*a*; red line) complex **5** (5 μM) in methanol; the interacting system containing **5** (5 μM)+TSQ (10 μM) in methanol: (*b*; blue line) immediately after preparation; (*c*; green line) 15 minutes after preparation; (*d*; purple line) 30 minutes after preparation; all showing the increasing intensity of the peak at *m/z* 719.18, corresponding to [Zn(TSQ)_2_-H]^+^. Other identified ionic species are marked by the arrows, together with the theoretical isotopic distribution of the associated ions shown in the insets.

**Figure 6:**
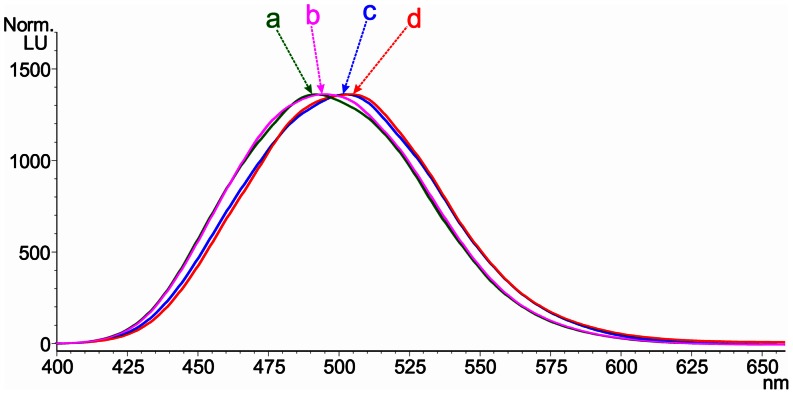
Fluorescence spectra of the interacting system containing either ZnSO_4_, or complex 5 and the used fluorescence probe. The fluorescence (emission spectra measured in the range of 400–900 nm with the excitation wavelength of 365 nm) spectra (the enlarged part 400–660 nm) of the interacting system: containing ZnSO_4_ (5 μM)+TSQ (10 μM) (*a*) immediately after preparation, arrow marks the λ_max_ at 492 nm; (*b*) 30 minutes after preparation, arrow marks λ_max_ at 494 nm; containing complex **5** (5 μM)+TSQ (10 μM) (*c*) immediately after preparation, arrow marks the λ_max_ at 503 nm; (*d*) 30 minutes after preparation, arrow marks λ_max_ at 508 nm. The excitation wavelength was 365 nm.

The use of TSQ in cytological fluorescent visualization of zinc(II) species was established in the early 1970s [48]. It has been used to detect and quantify “free” zinc(II) ions, as well as those bound to different biomolecules or cellular structures, such as lysosomal granules or neurons [49]–[51]. The usual concept of Zn and TSQ interaction presumes a 1∶2 metal-to-ligand ratio [52], [53]. Recent publications regarding the mechanistic studies of Zn-TSQ interactions, however, shed new light on this type of interaction in biological systems and confirm that in some conditions, the zinc(II) to TSQ ratio can be 1∶1 while the other two coordination positions are occupied by a biomolecule [54], [55]. Due to the above mentioned facts, it was unclear if the zinc(II) ions detected by fluorescence in the cell-uptake studies represented “free” zinc(II) ions or also zinc(II) ions transported into the cells in the form of zinc(II) complexes. To clarify this, at least theoretically, we analyzed the reaction system simulating the staining procedure for zinc by TSQ by ESI-MS. The mass spectrum of complex **5** in methanol, measured in the positive ionization mode, is dominated by the ions *m/z* 292.07 and 181.95, representing [L^5^+H]^+^ and its fragment [C_5_H_2_N_5_Cl = CH_2_+H]^+^, and contains three most intensive zinc-derived ions (shown here as the most intensive ions with a characteristic isotopic distribution) *m/z* 647.07 corresponding to the species [Zn(L^5^)_2_-H]^+^; *m/z* 682.97, corresponding to [Zn(L^5^)_2_Cl]^+^; and *m/z* 393.94, [Zn(L^5^)Cl]^+^. In the interacting systems (Figure 5), containing a mixture of complex **5** (final concentration of 5 μM) and TSQ (final concentration of 10 μM) in methanol, the time-dependent increase of intensity of the main zinc-containing ion at *m/z* 719.18, corresponding to the species [Zn(TSQ)_2_-H]^+^ was observed and accompanied by the adduct [Zn(TSQ)_2_-2H+Na]^+^ at *m/z* 741.13. In addition to this main ionic species, however, also the mixed-ligand species [Zn(TSQ)(L^5^)Cl-H]^+^ was observed at *m/z* 684.06. Coincidentally, this ion appears near the position where one of the ions from intact complex **5** can be found. In this case, however, the isotopic distribution pattern corresponds to the mixed-ligand species (see insets in the Figure 5). In addition to the mononuclear ionic species, those of higher nuclearity were observed in the mass spectra of the interacting systems, as confirmed by the presence of the ions at *m/z* 819.02 (corresponding to the ionic species [Zn_2_(L^5^)_2_Cl_3_]^+^), *m/z* 983.62, and at *m/z* 1113.02 ([Zn_2_(TSQ)_3_-3H]^+^). Another issue, accompanying the ESI-MS speciation was to confirm if there is any significant change in the fluorescence spectra of the interacting systems of **5** and TSQ that could, at least theoretically, impair the cytological visualization of zinc in the cell-uptake studies. Therefore, simultaneously with the ESI-MS analysis, we also performed a FIA-FLD analysis of the mixture of complex **5** (final concentration of 5 μM) and TSQ (final concentration of 10 μM; in two time periods, first immediately after mixing, and second after 30 minutes), compared with a reference sample, containing similar concentrations of ZnSO_4_ and TSQ. The fluorescence spectra (Figure 6) showed a significant difference in the peak maxima for the interaction system (λ_max_ = 503 nm, and 508 nm after 30 minutes, respectively) and the reference sample (λ_max_ = 492 nm, and 494 nm after 30 minutes, respectively). This red-shifting of the maxima is in direct contrast to the findings of Meeusen *et al*. (2011) [54], who described the 1∶1 ratio of zinc-to-TSQ in some zinc-containing biological samples. Nevertheless, even the changes in the fluorescence spectra caused by the reaction with complex **5** are not able to influence the cytological visualization of zinc, because they are not filtered out by the DAPI/FITC filters.

In an effort to assess the interactions of representative complex **5** with different relevant biomolecules (amino acids and small peptides), the electrospray-ionization mass-spectrometry (ESI-MS) measurements were carried out. The solutions of two most important sulfur-containing low-molecular biogenic compounds, containing physiological levels of cysteine (Cys, 290 μM) and reduced glutathione (GSH, 6 μM) [36], were studied in the mixture with 20 µM of complex **5**. The interacting system was analysed using ESI-MS spectrometry in the positive ionization mode. The measurements were performed in two time periods, the first measurement was carried out immediately after mixing of the three major components (cysteine+glutathione and complex **5**), while the second one was performed 30 minutes after mixing of the principal components. The results of both ESI-MS analyses were straightforward and unequivocally confirmed the formation of electroneutral complexes between zinc(II) and the sulfur-containing biomolecules, leaving the free kinetin-derived molecules in the solution. In both mass spectra only the ionic species, derived from the free L^5^ ligands were found (dominantly *m/z* 292.15, i.e. [L^5^+H]^+^) and those, representing the free biomolecules – cysteine (*m/z* 122.05, corresponding to [Cys+H]^+^) and reduced glutathione (*m/z* 308.08, corresponding to [GSH+H]^+^). No zinc containing ions were found in the mass spectra of the interacting systems.

### Conclusions

Our results indicate relatively low toxicity of complexes **1**–**7** and mixed pro- and anti-inflammatory activities. The pro-inflammatory effect is demonstrated by the slightly higher TNF-α secretion and lower pro-MMP-2/MMP-2 ratio and the anti-inflammatory potential is shown by significant diminishing of IL-1β secretion. These results justify further investigation of these complexes for their inflammation modulating potential *in vitro* and *in vivo*. Additionally, the studies on the interactions of the complexes with sulfur-containing biomolecules (l-cysteine and reduced glutathione) in biologically relevant concentrations, revealed high affinity of zinc(II) towards these biomolecules, when the ligand exchange was confirmed and free L^n^ molecules were detected by ESI-MS.
